# Supplementary material for: Sleep Disturbances and Phenoconversion in Patients with REM Sleep Behavior Disorder
Source: J Clin Med. 2021 Oct 14;10(20):4709. doi: 10.3390/jcm10204709 (PMC8536960; doi:10.3390/jcm10204709)
Supplement: Supplementary file 1 [file jcm-10-04709-s001.zip › jcm-1400108-supplementary.pdf]

Table S1. Correlation between RBD-related factors and sleep-related factors in patients with RBD (*n* = 226).

	Age at RBD Onset	RBD Duration	Frequency of DEB	Presence of Neurological Disorder	KVSS
ESS	−0.027	0.091	−0.574	0.383	−0.160
ISI	0.672	−0.465	−0.378	−0.166	0.041
PSQI	0.810 * (0.015)	−0.513	−0.551	−0.146	−0.463
K–BDI–II	−0.004	−0.616	−0.070	−0.076	0.311
TST	−0.136	0.583	0.055	−0.138	−0.324
SL	0.077	−0.745 * (0.034)	0.103	−0.333	0.315
REM latency	−0.313	0.460	−0.343	0.804 * (0.016)	−0.087
WASO, percent	0.115	−0.103	−0.316	0.517	0.021
N1	−0.192	0.477	−0.185	0.420	0.117
N2	0.384	−0.185	−0.522	0.058	−0.780 * (0.023)
N3	0.056	−0.183	0.844 * (0.008)	−0.614	0.620
REM	−0.245	−0.255	0.556	−0.348	0.587
AI	0.004	0.485	−0.307	0.202	−0.249
REM AI	−0.085	0.385	−0.590	0.229	−0.702

ESS, Epworth sleepiness scale; ISI, insomnia severity index; PSQI, Pittsburgh sleep quality index; BDI-II, Beck depression inventory-II; BMI, body mass index; WASO, wake after sleep onset; REM, rapid eye movement; NREM, non-rapid eye movement; AHI, apnea-hypopnea index; RDI, respiratory disturbance index; PLMI, periodic limb movements of sleep index; MAI, movement arousal index; RSWA, REM sleep without atonia. * *p*-value < 0.05.

Table S2. Initial and follow-up PSG data of 19 non-converters.

Variables	Initial PSG (*n* = 19)	Follow-Up PSG (*n* = 19)	*p*-Value
Age at PSG, years	62.1 ± 9.5	64.8 ± 9.6	<0.001 *
Self-reported sleep measures			
ESS	6.13 ± 3.3	5.07 ± 3.0	0.061
ISI	9.5 ± 6.4	7.2 ± 6.2	0.081
PSQI	6.2 ± 4.7	5.7 ± 3.0	0.824
K-BDI-II	11.7 ± 5.8	9.8 ± 4.9	0.420
Anthropometric and Polysomnographic data			
BMI, kg/m^2	24.8 ± 2.1	24.8 ± 2.6	0.711
NC, cm	39.5 ± 5.3	38.3 ± 2.6	0.631
Total sleep time, min	369.3 ± 79.6	342.6 ± 66.1	0.355
Sleep latency, min	12.5 ± 13.4	16.4 ± 18.6	0.251
REM latency, min	81.1 ± 54.6	109.3 ± 73.9	0.184
WASO, %	15.1 ± 15.0	17.3 ± 12.6	0.495
Sleep efficiency, %	81.6 ± 15.7	79.3 ± 13.1	0.469
Sleep stages			
N1 sleep, %	19.3 ± 12.4	22.7 ± 15.0	0.295
N2 sleep, %	59.5 ± 10.2	56.6 ± 12.4	0.421
N3 sleep, %	1.3 ± 3.0	3.8 ± 6.1	0.069
REM sleep, %	20.0 ± 9.2	17.3 ± 5.2	0.408
Arousal Index, /h	19.7 ± 12.4	19.2 ± 8.6	0.717
REM arousal index, /h	16.8 ± 9.0	19.0 ± 8.9	0.293
AHI, /h	13.2 ± 15.0	12.7 ± 10.5	0.514

REM AHI, /h	13.7 ± 13.8	12.7 ± 10.5	0.820
RDI, /h	15.4 ± 14.7	14.1 ± 10.7	0.936
REM RDI, /h	16.2 ± 13.5	13.9 ± 10.3	0.650
PLMI	30.6 ± 18.7	36.3 ± 25.3	0.612
MAI	0.8 ± 1.4	2.4 ± 4.6	0.058

ESS, Epworth sleepiness scale; ISI, insomnia severity index; PSQI, Pittsburgh sleep quality index; BDI-II, Beck depression inventory-II; BMI, body mass index; NC, neck circumference; WASO, wake after sleep onset; REM, rapid eye movement; NREM, non-rapid eye movement; AHI, apnea-hypopnea index; RDI, respiratory disturbance index; PLMI, periodic limb movements of sleep index; MAI, movement arousal index; RSWA, REM sleep without atonia. * p-value < 0.05. Variables were analyzed by a Wilcoxon sign rank test.
